# Does the Caesarean Section Impact on 11β HSD2 and Fetal Cortisol?

**DOI:** 10.3390/ijerph17155566

**Published:** 2020-08-01

**Authors:** Aneta Słabuszewska-Jóżwiak, Marta Włodarczyk, Krzysztof Kilian, Zbigniew Rogulski, Michał Ciebiera, Jolanta Szymańska-Majchrzak, Kornelia Zaręba, Jacek Krzysztof Szymański, Dorota Raczkiewicz, Grażyna Nowicka, Grzegorz Jakiel

**Affiliations:** 1 First Department of Obstetrics and Gynaecology, Centre of Postgraduate Medical Education, Żelazna 90 Street, 01-004 Warsaw, Poland; kornelia3@poczta.onet.pl (K.Z.); jkszymanski2@gmail.com (J.K.S.); grzegorz.jakiel1@o2.pl (G.J.); 2 Department of Biochemistry and Pharmacogenomics, Faculty of Pharmacy, Medical University of Warsaw, Banacha 1 Street, 02-097 Warsaw, Poland; marta.wlodarczyk@wum.edu.pl (M.W.); gnowicka@wum.edu.pl (G.N.); 3 Center for Preclinical Research, Medical University of Warsaw, Banacha 1 Street, 02-097 Warsaw, Poland; 4 Heavy Ion Laboratory, University of Warsaw, Pasteura 5a Street, 02-093 Warsaw, Poland; kilian@slcj.uw.edu.pl; 5 Radiochemistry for Medicine and Industry, Biological and Chemical Research Centre, University of Warsaw, Banacha 1 Street, 02-097 Warsaw, Poland; rogul@chem.uw.edu.pl; 6 Second Department of Obstetrics and Gynaecology, Centre of Postgraduate Medical Education, Cegłowska 80 Street, 01-809 Warsaw, Poland; michal.ciebiera@gmail.com; 7 Chair and Department of Biochemistry, Medical University of Warsaw, Banacha 1 Street, 02-097 Warsaw, Poland; jolanta.szymanska@wum.edu.pl; 8 Institute of Statistics and Demography, Collegium of Economic Analysis, SGH Warsaw School of Economics, Madalińskiego 6/8 Street, 02-513 Warsaw, Poland; dorota.bartosinska@gmail.com

**Keywords:** 11β-HSD 2 activity, umbilical cord blood cortisol, pregnancy, placenta, caesarean section

## Abstract

Purpose: Comparison of the activity of 11beta-hydroxysteroid dehydrogenase type 2 in the placenta and the umbilical cord blood cortisol level between caesarean sections with or without uterine contraction and vaginal delivery groups. Cortisol is the main stress hormone responsible for the normal adaptation of the neonate to extrauterine life. The disorders resulting from a dysfunction of the 11β-HSD 2–cortisol system can explain the higher risk of developing diseases in children born by caesarean section. Methods: 111 healthy, pregnant women in singular pregnancy at term of delivery were included into the study. The study comprised 11β-HSD 2 in placental tissue from 49 pregnant women delivering by elective caesarean section and 46 pregnant women delivering by vagina. In 16 cases of the elective caesarean section, regular uterine contractions were declared. Cortisol level was estimated in umbilical cord blood directly after delivery. Results: We found no statistically significant differences in the activity of 11β-HSD 2 in placentas delivered via caesarean sections (29.61 on average in elective caesarean sections and 26.65 on average in intrapartum caesarean sections) compared to vaginal deliveries (31.94 on average, *p* = 0.381), while umbilical cord blood cortisol in the elective caesarean sections group was significantly lower (29.86 on average) compared to the vaginal deliveries (55.50 on average, *p* < 0.001) and intrapartum caesarean sections (52.27 on average, *p* < 0.001). Conclusions: The model of placental 11β-HSD 2 activity and umbilical cord blood cortisol concentration seems to be significant in conditions of stress associated with natural uterine contractions in labour.

## 1. Introduction

Glucocorticoids play a key role in regulating foetal intrauterine growth, affecting the development of the blood vessels in the placenta and controlling the delivery of nutrients to the foetus [1], thus exerting an influence on the cardiovascular system, metabolism and normal foetal homeostasis [2]. The level of glucocorticosteroids is regulated systemically by the hypothalamic pituitary adrenal (HPA) axis but also by the local “pre-receptor” activity of the enzyme 11beta-hydroxysteroid dehydrogenase (11β HSD) [3] that converts active cortisol into inactive cortisone.

A normally developing pregnancy is a period of hyperactivity of the HPA axis as well as hypercortisolemia. As the pregnancy progresses, a gradual increase is found in serum cortisol levels of pregnant women, reaching 3 times higher values in the third trimester compared to non-pregnant women [4]. In the foetus, however, low concentrations of cortisol are present during the first two trimesters of pregnancy, with the exception of the period around gestational week 10 when an increase in this hormone is observed due to the activity of the newly formed foetal adrenal gland tissue. In the third trimester of pregnancy, the foetal cortisol concentration rises and reaches peak levels in the perinatal period, caused by the weakened placental metabolism of this hormone and its increased synthesis by the foetal adrenals [5]. The elevated cortisol values during pregnancy exert an anti-inflammatory and catabolic effect both in the pregnant woman and the foetus.

The placenta, apart from its transport function and gas exchange between the mother and the foetus is also an endocrine organ producing corticotropin-releasing hormone (CRH) in the second and third trimester of pregnancy that is identical to the hypothalamic form. Placental CRH stimulates both the maternal and foetal pituitary to release the adrenocorticotropic hormone (ACTH) which in turn stimulates the adrenal glands to produce cortisol. At the same time, placental CRH also induces ACTH receptor expression in the foetal adrenals [6]. Contrary to the typical negative feedback mechanism where high cortisol concentrations suppress the HPA axis, during pregnancy cortisol stimulates placental CRH synthesis [7,8] leading to hypercortisolemia in the serum of the pregnant woman. When the maternal cortisol enters the foetal circulation it has a suppressive effect on the foetal pituitary, thus leading to its reduced excretion. With the maturation of the placenta there is increased 11β-HSD enzyme activity converting the maternal cortisol to cortisone [9] as a result of which its levels in foetal circulation are reduced, in turn triggering foetal pituitary ACTH synthesis [10]. The above mechanisms remain the subject of research.

Glucocorticoids are hormones that play an essential role in the normal intrauterine development of the foetus and normal tissue and organ differentiation. In lung, glucocorticosteroids stimulate the production of surfactant-associated proteins and increase phospholipid synthesis by enhancing the activity of phosphatidylcholine and play role in regulation of pulmonary fluid metabolism in order to survive in extra-uterine environment after birth [10,11]. To speed up a fetus’s lung development, antenatal prophylactic corticosteroids are administrated before elective caesarean section at term [12,13] or at preterm birth [14]. This helps to prevent respiratory distress syndrome (RDS) and reduce an incidence of neonatal intensive care unit admissions.

A basic system protecting the foetus against excessive maternal cortisol is the placental enzyme 11beta-hydroxysteroid dehydrogenase type 2 (11β-HSD 2).

11β-HSD is an NADPH-dependent oxidoreductase highly expressed in key metabolic tissues. Two distinct 11β-HSD isoforms have been described: 11β-HSD 1 and 11β-HSD 2, which are encoded by different genes and differ in function, site in the body, cofactor and substrate affinity. 11β-HSD 1 is mainly present in the liver where it converts inactive cortisone into active cortisol while the 11β-HSD 2 isoform dominates in the placenta where it breaks down 80–90% of the maternal cortisol into cortisone, which is transferred through the umbilical cord into the foetal circulation [5]. Peripheral glucocorticosteroid metabolism alters depending on the age, coexisting obesity or other diseases [15,16]. It has been highlighted in numerous epidemiological studies that factors like long-term stress, anxiety, depression or asthma reduce the placental activity of 11β-HSD 2 [17,18] and lead to hypercortisolemia in the foetus, which may result in neurobehavioural disorders [19] and an increased risk of developing hypertension [20] or diabetes in children during the early school period.

Delivery through the maternal passage by natural forces alone subjects neonates to intrapartum stress and elevated concentrations of catecholamines and cortisol are found in their blood [21]. Elevated stress hormones levels in the perinatal period are key to obtaining maturation of the lungs [22] and adaptation of the circulatory system [23] of the infant to extrauterine life. A potential factor changing this state is completing the pregnancy without the stress of birth by way of a planned, elective caesarean section.

A caesarean section is the most commonly performed surgical procedure globally in women. Maternal, and neonatological consequences of its, still have been a subject of many studies.

Our study is aimed to compare the activity of 11beta-hydroxysteroid dehydrogenase type 2 in the placenta and the umbilical cord blood cortisol level between caesarean sections with or without uterine contraction and vaginal delivery groups. Cortisol is the main stress hormone responsible for the normal adaptation of the neonate to extrauterine life. The disorders resulting from a dysfunction of the 11β-HSD 2–cortisol system can explain the higher risk of developing diseases like hypertension, diabetes or obesity in children born by caesarean section.

## 2. Materials and Methods

### 2.1. Study Group

The study was carried out in the First Clinic of Obstetrics and Gynaecology of the Medical Centre of Postgraduate Education in Warsaw. The study covered 111 healthy, pregnant Caucasian women admitted to the Department of Gynaecology in the hours of 10:00 a.m. to 1:30 p.m. with regular contractions or to have a planned caesarean section. Additional women meeting the criteria set out hereunder were included in the study group.

On the day of admission to the Department, the patients completed a survey, an anthropometric questionnaire and gave their written consent to participate in the study. Placental tissue samples were collected and umbilical cord blood samples were taken and secured for scientific purposes. Information regarding the height and body weight before pregnancy, the weight gain in pregnancy, the medications being taken, and the course of the pregnancy were obtained from the maternity card. In the survey, they were asked about their past diseases, operations, stimulants like cigarette smoking, alcohol, and drugs, as well as any medications taken, including antibiotics.

Pregnant women over 18 years of age with a singleton pregnancy at or above after week 37 of gestation were included in the study.

The exclusion criteria constituted multiple pregnancies, diagnosed diabetes (including diabetes in pregnancy), arterial hypertension (diagnosed before or during pregnancy), renal insufficiency, hepatic diseases or cholestasis and the presence of autoimmune diseases, respiratory diseases (asthma, obstructive pulmonary disorder), psychiatric diseases (depression, schizophrenia, and neurosis) and taking antibiotics less than 2 weeks before delivery or glucocorticosteroids. Furthermore, women hospitalised during pregnancy due to the risk of premature birth with a cervical cerclage or pessary, smoking tobacco products, and consuming alcohol or drugs were excluded too. The exclusion criteria also included disorders of placentation.

Women who gave birth to infants weighing less than 2500 g or whose neonates had an Apgar score of less than 7 points at 1 min and 5 min after birth, or required admission to a Neonatal Intensive Care Unit within the first 24 h after birth, or were diagnosed with a birth defect (including chromosomal aberration) were also excluded from the analyses.

None of the pregnancies resulted from assisted reproduction technology treatment. None of the deliveries were preceded by an oxytocin infusion to induce labour, the administration of prostaglandins or the premature rupture of membranes.

In 49 cases, the pregnancy was completed by delivery by elective caesarean section due to a preterm breech presentation, cephalopelvic disproportion or a previous caesarean section. In 16 cases, the operative delivery was preceded by the onset of regular contractions. Spinal (subarachnoid) anaesthesia was administered in all operative deliveries. It should be emphasised that no caesarean section was carried out under general anaesthesia or due to the risk of intrauterine foetal asphyxia or the lack of progress in delivery.

The control group comprised 46 pregnant women whose delivery was completed through the maternal passage by natural forces alone. In 31 cases, epidural anaesthesia was administered to relieve pain during labour. Operative vaginal births with the use of an obstetric vacuum extractor (ventouse) or obstetrical forceps were excluded from the study.

The study was approved by the Ethics Committee of the Postgraduate Medical Education Centre in Warsaw (No. 8/PB/2014).

### 2.2. Determination of 11β-HSD 2 and Cortisol

In order to determine 11β-HSD 2 activity, placental tissue was collected within 45 min of delivery. In all cases, the delivery of the placenta was natural. The placenta was excluded from further research if placental tissue defects were found or in the event of its manual extraction. Full-thickness placental tissue fragments were collected in compliance with aseptic technique and with sterile instruments. All tissue specimens were obtained close to the umbilical cord insertion site to the placenta.

The tissue was subsequently divided into three horizontal segments from the basal towards the chorionic surface. Each sample was rinsed thoroughly in a cold sterile saline solution (PBS, pH 7.5), and the samples from the different sections were snap frozen in liquid nitrogen and stored at −80 °C for protein analysis.

The cord blood was processed by the laboratory within 20 min, centrifuged, and the sera were aliquoted and stored at −20 °C until assayed.

The frozen placental tissue (about 100 mg) was homogenized on ice for 30 sec with a mechanical homogenizer (Omni International Kennesaw, GA, USA) in ice-cold 0.1 M PBS pH 7.5 supplemented with anti-proteases (Sigma-Aldrich Saint Louis, MO, USA). The tissue homogenate was centrifuged at 10,000× *g* for 30 min at 4 °C. The resulting supernatant was collected and assayed for protein concentration using Qubit 2.0. (ThermoFisher Scientific Waltham, MA, USA) and the Qubit Protein Assay Kit (Thermo Fisher Scientific, Waltham, MA, USA). To measure the activity of 11β-HSD-2, human placental homogenates (0.4 mg of protein) were incubated in a 1 mL phosphate buffer (0.1 M, pH 7.6) in the presence of 17 kBq (1,000,000 dpm) of [1,2,6,7-^3^H]-cortisol, 0.25 µM of cortisol and 5 µM of cofactor NAD^+^ for 30 min at 37 °C in a dry heater with shaking. Samples were extracted with dichloromethane (10 × 0.5 mL), collected and evaporated using an inert argon gas stream in a dry heater at 50 °C. Each sample of steroids were dissolved in 0.5 mL of methanol and separated by Reversed-Phase High Performance Liquid Chromatography (RP-HPLC) with UV detection at λ = 240 nm, using isocratic elution with a methanol-water mixture (55:45, *v/v*) as a mobile phase (0.9 mL/min flow, oven temperature: 27 °C, column C8 250 mm × 4.6 mm × 5 µm). The radioactivity of collected fractions containing tritium-labelled cortisol or cortisone were measured by liquid scintillation using an organic scintillation cocktail. The specific activity of 11β-HSD-2 was expressed as the amount of cortisone (in picograms) formed per minute per mass of protein (in mg).

Tritiated [1,2,6,7-^3^H]-cortisol was from PerkinElmer (Waltham, MA, USA), cortisol, cortisone and NAD^+^ were from Sigma-Aldrich, Saint Louis, MO, USA). Other chemicals like dichloromethane was from POCH S.A. (Gliwice, Poland) and methanol (HPLC grade Lichrosolv) was from Merck (Darmstadt, Germany) Universal scintillation cocktail used in studies was Rotiszint eco plus.

Steroids were separated using HPLC Shimadzu LC20AD with SPDM20A Diode Array Detection (Shimadzu, Canby, OR, USA.). Radioactivity was measured by liquid scintillation counter Tri-Carb 2910TR from PerkinElmer.

Cord blood cortisol was determined using a commercial ELISA kit (LDN Labor Diagnostika Nord GmbH & Co. KG, Nordhorn, Germany). The sensitivity of this assay is 0.4 μg/dL. The intra- and inter-assay coefficient of variations are 5.8 and 5.6% respectively.

### 2.3. Statistical Analyses 

The data were statistically analysed using STATISTICA 13 software (StatSoft Inc., Cracow, Poland). Minimum and maximum values and the median and interquartile range (lower (25%) and upper (75%) quartiles) were estimated for continuous variables, as well as absolute numbers (*n*) and percentages (%) of the occurrence of items for categorical variables.

The following statistical tests were used:

− Kruskal-Wallis’ H test to compare cortisol concentration and 11β-HSD 2 between three modes of birth;
− Mann-Whitney’s test to compare cortisol concentration and 11β-HSD 2 between two modes of birth in pairs, between male and female neonates, as well as between epidural analgesia and no analgesia in vaginal deliveries;
− Spearman’s correlation coefficient (r) to correlate: cortisol concentration and 11β-HSD 2, cortisol concentration and continuous characteristics, 11β-HSD 2 and continuous characteristics.

The significance level was taken to be 0.05.

## 3. Results

The characteristics of the study group including maternal and gestational age, parity, pre-pregnancy and pregnancy BMI, weight gain during pregnancy, newborn weight, height, and sex, Apgar scores at both 1st and 5th min, applied analgesia in three modes of birth as well as the duration of uterine contraction in vaginal deliveries, and is presented in Table 1. The same study group’s characteristics was presented in our previous study, but that study was aimed to compare global DNA methylation in placenta between caesarean sections with or without uterine contraction and vaginal deliveries [24].

The uterine contractions lasted from 1 to 12 h in vaginal deliveries (5.5 h on average). Spinal analgesia was administered in all the women delivering by caesarean sections, while epidural analgesia in 67% of the vaginal deliveries.

Placental 11β-HSD 2 did not significantly differ between the three modes of birth (H = 1.930, *p* = 0.381). It was estimated at 29.61 on average in the elective caesarean sections, at 26.65 on average in the intrapartum caesarean sections, and at 31.94 on average in the vaginal deliveries (Figure S1).

Umbilical cord blood cortisol significantly differed between three modes of delivery groups (H = 37.148, *p* < 0.001). In the elective caesarean sections, umbilical cord blood cortisol was significantly lower (29.86 on average) compared to the intrapartum caesarean sections (52.27 on average, *p* < 0.001), and in relation to the vaginal deliveries (55.50 on average, *p* < 0.001). This cortisol did not significantly differ between the intrapartum caesarean sections and the vaginal deliveries (*p* = 0.573; Figure S2).

No correlation was found between placental 11β-HSD 2 and umbilical cord blood cortisol in the elective caesarean sections (r = 0.169, *p* = 0.246) and in the intrapartum caesarean sections (r = −0.129, *p* = 0.633). A negative correlation was found between placental 11β-HSD 2 and umbilical cord blood cortisol in the vaginal deliveries (r = −0.502, *p* < 0.001; Figure S3).

Placental 11β-HSD 2 correlated negatively with pregnancy BMI (r = −0.361, *p* = 0.011) and with Apgar score at 5th minute (r = −0.336, *p* = 0.018) in elective caesarean section group. Placental 11β-HSD 2 did not correlate with maternal and gestational age, parity, weight gain during pregnancy and pre-pregnancy BMI, newborn weight and gender, or with Apgar score at 1st minutes in elective caesarean section group. No correlation was found between placental 11β-HSD 2 and characteristic in intrapartum caesarean section and vaginal delivery groups (Table 2).

Umbilical cord blood cortisol correlated negatively with Apgar score at 5th minute in elective caesarean section group (r= −0.342, *p* = 0.016) and positively with duration of uterine contraction in vaginal delivery group (r = 0.369, *p* = 0.012). However, umbilical cord blood cortisol did not correlate with other characteristics in different modes of delivery groups (Table 3).

Placental 11β-HSD 2 in female newborns was significantly higher (32.89 on average) than in the male newborns (26.59 on average) in the total study group (*p* = 0.018), but umbilical cord blood cortisol did not significantly differ between the gender of the neonates (*p* = 0.118), (Figure S4).

## 4. Discussion

Placental 11β-HSD 2 is one of the factors regulating foetal serum cortisol. In our study, we found no statistically significant differences in the activity of 11β-HSD 2 in placentas delivered via caesarean sections compared to vaginal deliveries, while umbilical cord blood cortisol levels differed significantly across the different modes of delivery. In the case of elective, operative deliveries, the umbilical cord blood cortisol levels were significantly lower (29.86) compared to vaginal deliveries (55.50 on average, *p* < 0.001) Despite significant positive correlation between umbilical cord blood cortisol and duration of uterine contraction, 11β-HSD 2 was not associated with mode of delivery. Furthermore, we observed a negative correlation between the activity of placental 11β-HSD 2 and umbilical cord blood cortisol, however, this phenomenon was significant only in the unassisted vaginal delivery group. Our observation supports the hypothesis that cortisol acts the main role of infant adaptation to extrauterine life.

Numerous studies concern the impact of stress on the maternal and foetal salivary, serum, amniotic cortisol and cortisone levels [17,25,26]. If the maternal cortisol level was compared to amniotic fluid cortisol, this indicated low placental 11β-HSD 2 activity. Ghaemmaghami demonstrated a positive correlation between acute, short-term stress during pregnancy and the activity of the placental dehydrogenase enzyme [17]. It seems that delivery by the natural forces of labour and the effort that it entails is the situation that triggers the system mediating 11β-HSD 2–cortisol regulation, which should result in a lower level of cortisol from the mother. In our study, newborns delivered through the maternal passage by natural forces had significantly higher levels of cortisol accompanied by a negative correlation between 11β-HSD 2 and cortisol that may, on the one hand, demonstrate perinatal stimulation of the foetal adrenal glands resulting in an increase in cortisol but, on the other, point to the functioning of the 11β-HSD 2–cortisol complex, which is indicative of the possible presence of maternal cortisol in the assessed pool of cord blood.

The impact of the uterine contractile action on 11β-HSD 2 activity and cortisol levels is interesting. As for the presence of uterine contractile activity preceding an operative delivery, the cortisol level was significantly higher (52.27, on average, *p* < 0.001) compared to the operative deliveries without the contractile action of the uterus (29.86 on average). It is noteworthy that the occurrence of contractile activity remained without significant influence on 11β-HSD 2 activity and only affected the cortisol level. Dahlerup and colleagues introduced the concept of adjusted foetal cortisol exposure (AFCE) showing the differences between the maternal and foetal cortisone to cortisol ratio, which may indirectly be indicative of the activity of 11β-HSD 2 in the placenta. The results of their research indicate the increased activity of the enzyme during a natural delivery because AFCE was significantly lower after a caesarean section delivery [25]. In our research we determined the activity of 11β-HSD 2 directly and found no differences depending on the mode of delivery, hence, the higher level of cortisol in the umbilical cord blood after a delivered by the natural passages may result from both its elevated maternal serum level and increased foetal adrenal activity. The above results may suggest that contractile activity is an independent stress stimulus activating perinatal mechanisms that increase the cortisol levels in the infant, which are responsible for proper adaptation to extrauterine life. In the study conducted by us, we failed to show statistically significant differences in cortisol concentrations in operative deliveries with uterine contractions and deliveries by the natural passages. Thus, it is reasonable to assume that uterine contractile action is a factor influencing the level of cortisol and not merely the fact of completing the pregnancy by way of caesarean section.

There are many studies evaluating 11β-HSD 2 activity and the level of cortisol in different disease entities accompanying pregnancy, for example, pre-eclampsia or foetal hypotrophy [26,27], and taking into account the influence of stress or anxiety [28] but only a few of them concern physiological pregnancies and the mode of delivery. Many authors emphasize that both the mode of delivery and the type of anaesthesia administered are associated with the foetal hormonal stress response [29,30,31,32,33,34]. Schuller- and colleagues found that newborns delivered through the maternal passage by natural forces are characterised by higher cortisol levels and demonstrate a higher pain expression compared to children born by caesarean section [35]. Other researchers claim, however, that cortisol levels correlate positively with the duration of the second stage of labour as well as an operative completion of the pregnancy with the use of an obstetric vacuum extractor (ventouse) [34]. In our analyses, we failed to demonstrate significant differences in the activity of 11β-HSD 2 and cortisol in the case of the administration of extradural anaesthesia to relieve labour pain in the natural delivery group.

The 11β-HSD 2 enzyme is treated as the main barrier protecting the foetus against very high levels of maternal cortisol [36] and the consequences resulting from this phenomenon. Various factors may affect the activity of this enzyme like hypoxia [37], heavy metals like cadmium found in tobacco smoke [38] or the stimulation of the MAPK pathway [39]. In our study, we have demonstrated the negative impact of maternal BMI on the activity of this enzyme (r = −0.361, *p* = 0.011) in elective caesarean group, which may contribute to the intrauterine exposure of the foetus to glucocorticosteroids, thus leading to disruptions in intrauterine foetal programming. In an animal model, it was shown that obesity and ahigh-calorie maternal diet impacts offspring behaviour and their physiology [40]. In humans, long-term intrauterine exposure to glucocorticosteroids, including cortisol leads to foetal programming changes [41] and, in consequence, to the development of cardiometabolic diseases [42] or obesity in adult life [43]. With the progress of pregnancy, physiologic increments of cortisol occur in the maternal blood, which are higher in primi gravida [44]. Maternal age, pre-pregnancy BMI [45], tobacco smoking, and high body weight [46] also influence the change in its concentration. Our observations confirm that gestational age, pre- pregnancy BMI and percentage weigh gain during pregnancy significant change umbilical cord blood cortisol level, however these changes were not find between different mode of delivery. Moreover, we have demonstrated the significant impact of mode of delivery and duration of uterine contraction on the cortisol levels in umbilical blood. This illustrates the multifactorial aspects of cortisol levels regulation in pregnancy.

Our finding are consistent with literature and showing 11β-HSD 2 enzyme sexual dimorphism. A significantly higher level of dehydrogenase activity was observed in the placentas of female neonates compared to the placentas of male newborns (*p* = 0.018) in the total study group. The above results have been confirmed in animal and human models [47,48]. Murphy and colleagues not only concluded a higher dehydrogenase activity in girls but also demonstrated that in cases of maternal asthma, female foetuses demonstrate a lower activity of this enzyme while in male foetuses the levels remain unchanged. Despite the differences in 11β-HSD 2 activity, the cord blood cortisol levels were similar in both sexes [49]. Our findings demonstrate a lower cortisol concentrations in the umbilical cord blood of girls than in boys but the differences were not statistically significant. Hence, it is possible to deduce that female foetuses are more vulnerable to the effects of environmental factors reducing 11β-HSD 2 activity, which may have implications on the development of certain diseases, including depression [50]. However, the sexual dimorphism of 11β-HSD 2 activity was not found in modes of deliveries analysed separately. The matter concerning intrauterine sexual dimorphism, placental differences and their possible consequences still remains in the sphere of research and conjecture.

A possible limitation of our study is that all cord blood samples were collected between 10:00 a.m. and 1.30 p.m. Serón–Ferré and colleagues reported that cortisol levels of cord blood had a clear diurnal rhythm with the acrophase in the afternoon [51]. This finding suggested that foetuses have an adrenal circadian rhythm, which is entrained in antiphase to maternal rhythm. Additionally, a possible limitation of our study is the absence of determination of perinatal maternal cortisol. Data from literature show that there are higher cortisol levels in the serum of mothers giving birth naturally. Lower cortisol levels in neonates delivered by caesarean section may explain the higher rate of adaptation disorders. It should be emphasised that our study covered newborns from physiological pregnancies, carried to full term, born in an overall good condition, and not requiring hospitalisation in a Neonatal Intensive Care Unit; nevertheless, everything showed significantly lower levels of cortisol in the umbilical cord blood of children delivered by elective caesarean section. Hence the question concerning the relevancy of administering glucocorticosteroids before an elective caesarean section still remains open. A limitation of our study is a small sample size of the caesarean section group preceded by regular uterine contractions, which would fully confirm the influence of this factor on cortisol concentration.

## 5. Conclusions

The model of placental 11β-HSD 2 activity and umbilical cord blood cortisol concentration studied by us seems to be significant in conditions of stress associated with natural uterine contractions in labour; in the case of planned caesarean sections, there is no evidence supporting its significance. Despite the proven impact of various factors on placental 11β-HSD 2 activity, in conditions of the absence of labour stress, the activity of this enzyme does not change umbilical cord cortisol level.

## Figures and Tables

**Table 1:** Clinical characteristics of the study group.

Characteristics	Total Group (*N* = 111)	Elective Caesarean Section (*n* = 49)	Intrapartum Caesarean Section (*n* = 16)	Vaginal Delivery (*n* = 46)	*p* ^#^
Maternal age (years), Me (25–75%)	31 (28–35)	32 (30–36)	30 (28–36)	30 (28–33)	0.085
Gestational age (weeks), *n* (%)					0.004
37	11 (9.91)	5 (10.20)	1 (6.25)	5 (10.87)	
38	18 (16.22)	8 (16.33)	4 (25.00)	6 (13.04)	
39	51 (45.95)	31 (63.27)	4 (25.00)	16 (34.78)	
40	18 (16.22)	3 (6.12)	2 (12.50)	13 (28.26)	
41	13 (11.71)	2 (4.08)	5 (31.25)	6 (13.04)	
Parity, *n* (%)					0.122
1	63 (56.76)	22 (44.90)	13 (81.25)	28 (60.87)	
2	42 (37.84)	25 (51.02)	3 (18.75)	14 (30.43)	
3	5 (4.50)	2 (4.08)	0 (0.00)	3 (6.52)	
4	1 (0.90)	0 (0.00)	0 (0.00)	1 (2.17)	
Pre-pregnancy BMI (kg/m^2^), Me (IQR)	23.12 (20.81–25.46)	24.21 (21.51–26.70)	21.62 (19.47–25.06)	22.75 (20.70–24.91)	0.092
Pregnancy BMI (kg/m^2^), Me (IQR)	28.63 (26.03–30.82)	29.14 (27.28–30.80)	27.42 (25.71–31.71)	28.26 (25.82–30.45)	0.297
Weight gain during pregnancy (kg), Me (IQR)	14 (11–18)	14 (11–18)	17 (14–19)	14 (11–17)	0.494
Weight gain during pregnancy (%), Me (IQR)	21.92 (16.67–29.03)	21.67 (15.15–27.59)	30.20 (19.09–33.91)	22.02 (16.92–28.57)	0.203
Newborn weight (kg), Me (IQR)	3.52 (3.25–3.85)	3.50 (3.32–3.85)	3.62 (3.41–3.80)	3.51 (3.12–3.85)	0.476
Newborn gender, *n* (%)					0.356
male	51 (45.95)	21 (42.86)	10 (62.50)	20 (43.48)	
female	60 (54.05)	28 (57.14)	6 (37.50)	26 (56.52)	
Apgar score at 1st minute, *n* (%)					0.065
7	1 (0.90)	0 (0.00)	0 (0.00)	1 (2.17)	
8	7 (6.31)	6 (12.24)	1 (6.25)	0 (0.00)	
9	17 (15.32)	11 (22.45)	2 (12.50)	4 (8.70)	
10	86 (77.48)	32 (65.31)	13 (81.25)	41 (89.13)	
Apgar score at 5th minute, *n* (%)					0.188
7	1 (0.90)	0 (0.00)	0 (0.00)	1 (2.17)	
8	1 (0.90)	1 (2.04)	0 (0.00)	0 (0.00)	
9	10 (9.01)	8 (16.33)	0 (0.00)	2 (4.35)	
10	99 (89.19)	40 (81.63)	16 (100.00)	43 (93.48)	
Duration of uterine contraction (hours), Me (IQR)	-	-	-	5.46 (3.50–7.67)	-
Analgesia *, *n* (%)	96 (86.49)	49 (100.00)	16 (100.00)	31 (67.39)	-

Me—median, IQR—interquartile range (25–75%). * spinal for caesarean section or epidural for vaginal delivery, ^#^ Kruskal-Wallis’ H test or χ2 test were used to compare continuous or categorical variables respectively, between 3 modes of birth.

**Table 2:** Correlations between placental 11β-HSD 2 and maternal or offspring outcomes.

Characteristics	Method ^#^	Total Group (*n* = 111)		Elective Caesarean Section (*n* = 49)		Intrapartum Caesarean Section (*n* = 16)		Vaginal Delivery (*n* = 46)	
		Test	*p*	Test	*p*	Test	*p*	Test	*p*
Maternal age (years)	r	−0.073	0.447	−0.119	0.415	0.251	0.348	−0.085	0.575
Gestational age (weeks)	r	0.193	0.042	0.099	0.499	0.322	0.224	0.187	0.214
Parity	r	0.036	0.710	0.047	0.749	0.122	0.654	0.042	0.781
Pre-pregnancy BMI (kg/m^2^)	r	−0.091	0.343	−0.168	0.248	0.107	0.692	−0.004	0.981
Pregnancy BMI (kg/m)	r	−0.198	0.037	−0.361	0.011	−0.031	0.910	−0.079	0.600
Weight gain during pregnancy (kg)	r	−0.155	0.105	−0.160	0.273	−0.243	0.365	−0.113	0.155
Weight gain during pregnancy (%)	r	−0.117	0.220	−0.061	0.679	−0.311	0.242	−0.124	0.411
Newborn weight (kg)	r	−0.059	0.592	−0.008	0.956	0.015	0.957	−0.073	0.628
Newborn gender	Z	2.358	0.018	1.606	0.108	1.030	0.303	1.185	0.236
Apgar at 1st minute	r	−0.006	0.951	−0.170	0.243	−0.188	0.486	0.175	0.245
Apgar at 5th minute	r	−0.086	0.367	−0.336	0.018	na	-	0.195	0.195
Epidural analgesia *	Z	-	-	-	-	-	-	0.047	0.963
Duration of uterine contraction (hours) *	r	-	-	-	-	-	-	−0.228	0.127

* only in vaginal deliveries. ^#^ r—Spearman’s correlation coefficient, Z—Mann-Whitney’s test. na—not applicable because all newborns delivered by intrapartum caesarean section had the same Apgar at 5th min scored at 10.

**Table 3:** Correlations between umbilical cord blood cortisol and maternal or offspring outcomes.

Characteristics	Method ^#^	Total Group (*n* = 111)		Elective Caesarean Section (*n* = 49)		Intrapartum Caesarean Section (*n* = 16)		Vaginal Delivery (*n* = 46)	
		Test	*p*	Test	*p*	Test	*p*	Test	*p*
Maternal age (years)	r	−0.176	0.065	−0.178	0.222	−0.227	0.397	0.160	0.288
Gestational age (weeks)	r	0.192	0.044	0.123	0.400	0.490	0.054	−0.080	0.597
Parity	r	−0.145	0.129	0.177	0.222	−0.052	0.848	−0.105	0.489
Pre-pregnancy BMI (kg/m^2^)	r	−0.198	0.037	−0.155	0.288	0.025	0.927	−0.015	0.922
Pregnancy BMI (kg/m)	r	−0.116	0.227	−0.194	0.181	0.102	0.708	0.069	0.650
Weight gain during pregnancy (kg)	r	0.131	0.172	0.094	0.520	0.083	0.759	0.156	0.300
Weight gain during pregnancy (%)	r	0.207	0.029	0.117	0.425	0.255	0.341	0.205	0.171
Newborn weight (kg)	r	−0.005	0.955	−0.003	0.065	−0.239	0.374	0.119	0.430
Newborn gender	Z	−1.565	0.118	−0.182	0.237	−0.380	0.704	−0.543	0.587
Apgar at 1st minute	r	0.031	0.749	−0.246	0.089	−0.071	0.793	−0.066	0.664
Apgar at 5th minute	r	−0.058	0.543	−0.342	0.016	na	-	−0.125	0.408
Epidural analgesia *	Z	-	-	-	-	-	-	1.265	0.206
Duration of uterine contraction (hours) *	r	-	-	-	-	-	-	0.369	0.012

* only in vaginal deliveries. ^#^ r—Spearman’s correlation coefficient, Z—Mann-Whitney’s test. na—not applicable because all newborns delivered by intrapartum caesarean section had the same Apgar at 5th min scored at 10.

## Supplementary Materials

The following are available online at https://www.mdpi.com/1660-4601/17/15/5566/s1, Figure S1: Placental 11β-HSD 2 in three modes of delivery. Notes: *p* for Mann-Whitney’s test. The numerical values next to the boxes represent median. Figure S2: Umbilical cord blood cortisol in three modes of delivery. Notes: *p* for Mann-Whitney’s test. The numerical values next to the boxes represent median. Figure S3: Correlation between placental 11β-HSD 2 and umbilical cord blood cortisol in three modes of delivery. Notes: r—Spearman’s correlation coefficient. Figure S4: Placental 11β-HSD2 and umbilical cord blood cortisol versus newborn gender. Notes: *p* for Mann-Whitney’s test. The numerical values next to the boxes represent median.
