# Supplementary material for: Does the Caesarean Section Impact on 11β HSD2 and Fetal Cortisol?
Source: Int J Environ Res Public Health. 2020 Aug 1;17(15):5566. doi: 10.3390/ijerph17155566 (PMC7432821; doi:10.3390/ijerph17155566)
Supplement: Supplementary file 1 [file ijerph-17-05566-s001.pdf]

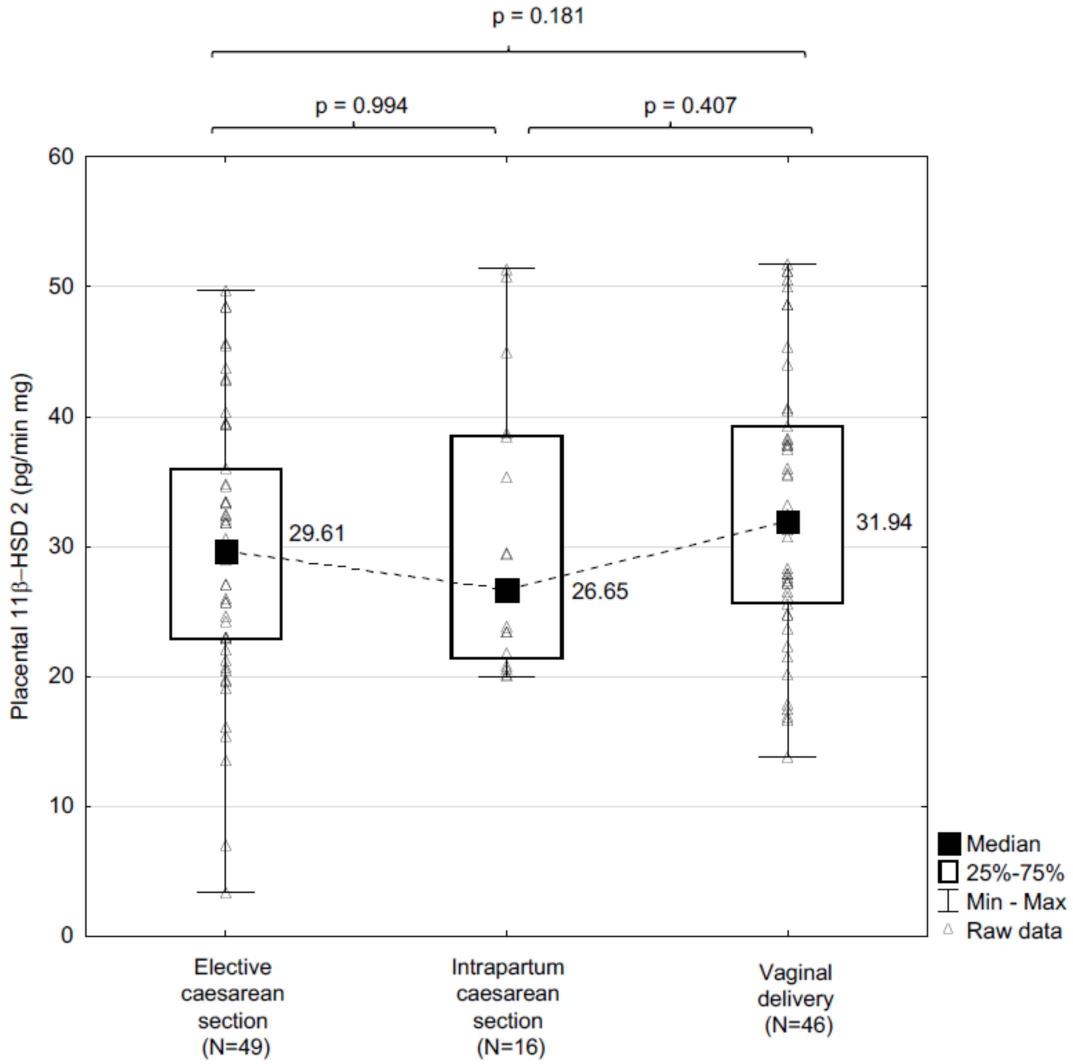

Figure S1. Placental 11β-HSD 2 in three modes of delivery. Notes: *p* for Mann-Whitney's test. The numerical values next to the boxes represent median.

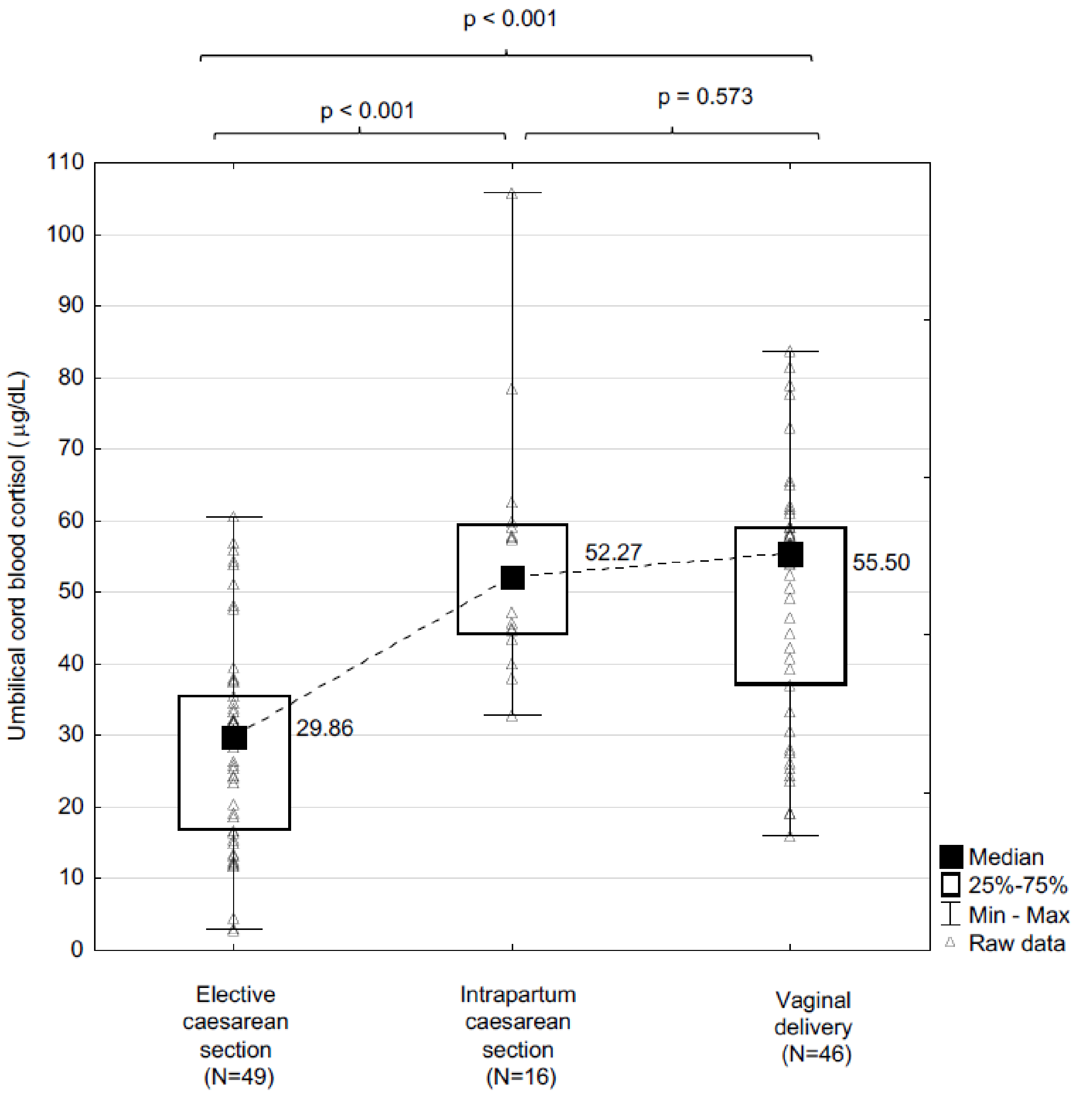

Figure S2. Umbilical cord blood cortisol in three modes of delivery. Notes: *p* for Mann-Whitney's test. The numerical values next to the boxes represent median.

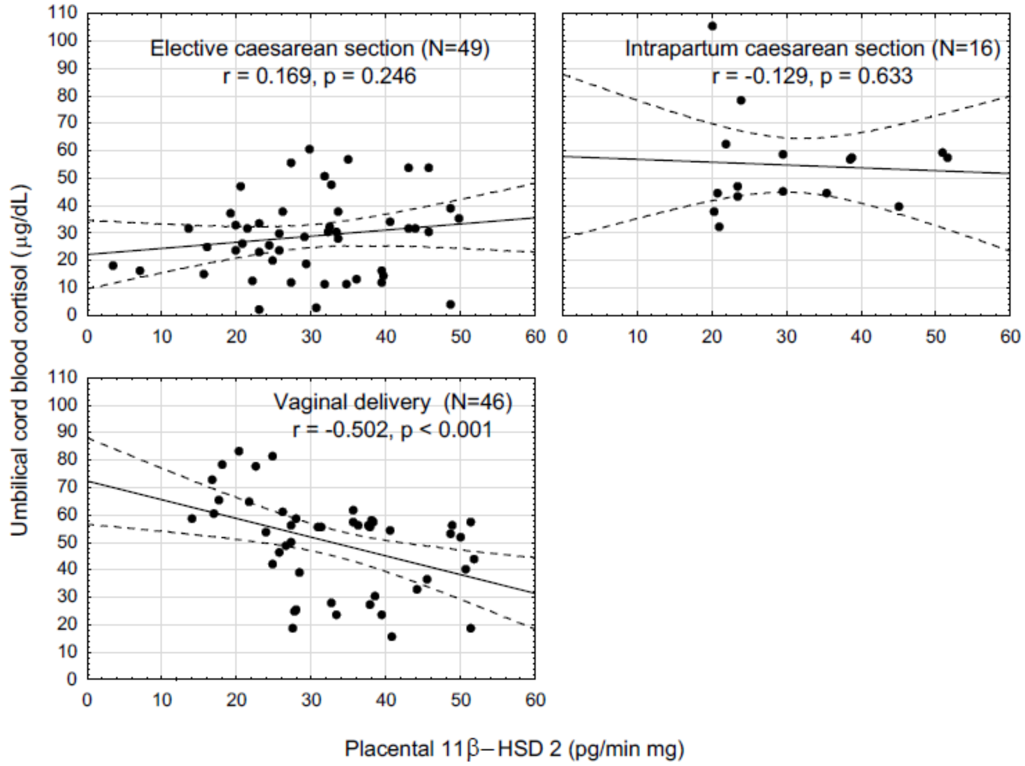

Figure S3. Correlation between placental 11β-HSD 2 and umbilical cord blood cortisol in three modes of delivery. Notes: r—Spearman's correlation coefficient.

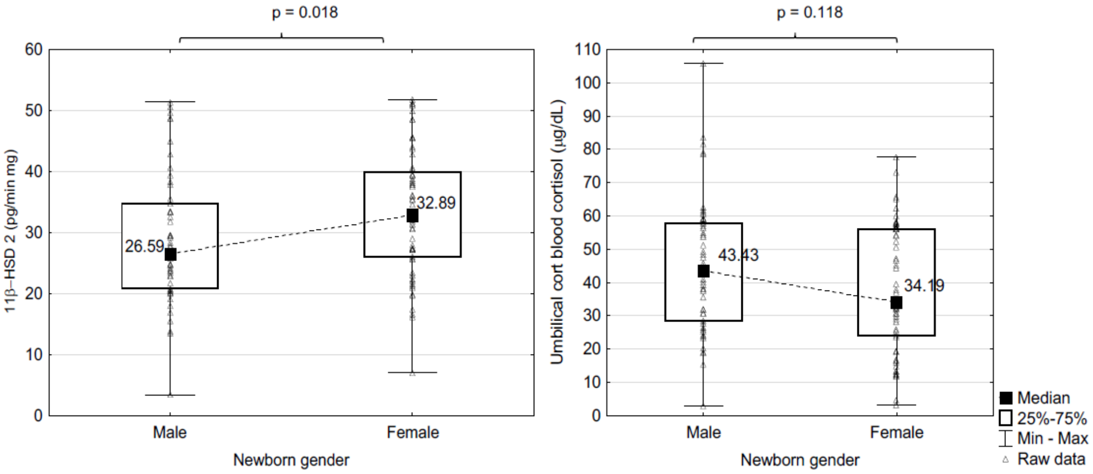

Figure S4. Placental 11β-HSD2 and umbilical cord blood cortisol versus newborn gender. Notes: *p* for Mann-Whitney's test. The numerical values next to the boxes represent median.
